# Effect of porcine immature oocyte vitrification on oocyte-cumulus cell gap junctional intercellular communication

**DOI:** 10.1186/s40813-020-00175-x

**Published:** 2020-11-25

**Authors:** Fahiel Casillas, Yvonne Ducolomb, Alma López, Miguel Betancourt

**Affiliations:** 1 Departamento de Biología de la Reproducción, División de Ciencias Biológicas y de la Salud, Universidad Autónoma Metropolitana-Iztapalapa, 09340 CDMX, México; 2 Departamento de Ciencias de la Salud, División de Ciencias Biológicas y de la Salud, Universidad Autónoma Metropolitana-Iztapalapa, 09340 CDMX, México

**Keywords:** Vitrification, Porcine, Oocyte, Viability, Maturation, Gap junctions

## Abstract

Vitrification may severely affect cumulus cells and oocyte morphology and viability, limiting their maturation and developmental potential. The aim of this study was to evaluate the gap junction intercellular communication (GJIC) integrity after the vitrification of porcine immature cumulus-oocyte complexes (COCs). Fresh COCs were randomly distributed in three groups: untreated (control), toxicity (cryoprotectants exposure), and vitrification, then subjected to in vitro maturation (IVM). Oocyte viability and IVM were measured in all groups. The evaluation of GJIC was expressed as relative fluorescence intensity (RFI). Vitrification significantly decreased oocyte viability and maturation after 44 h of culture compared to control. Also, significantly reduced RFI was observed in vitrified COCs during the first hours of culture (4–8 h) compared to control. This study demonstrates that porcine oocyte viability and maturation after 44 h of culture decreased after vitrification. GJIC was also affected during the first hours of culture after the vitrification of immature oocytes, being one of the possible mechanisms by which oocyte maturation decreased.

## Introduction

Oocyte cryopreservation has enabled significant advances in fertility treatments in humans and allows the genetic improvement of livestock. It has been reported that vitrification may severely affect cumulus cells (CCs) and oocyte morphology and viability, limiting their maturation and developmental potential [1]. Premature meiotic progression [2], low maturation [3, 4], and embryo development rates [5, 6] have been reported after the vitrification of porcine germinal vesicle (GV) immature oocytes. So far, some studies have reported the birth of live offspring from vitrified GV oocytes [7], and porcine embryos [8, 9].

Oocyte growth is related to the functionality of the somatic cells surrounding the oocyte, which are known as granulosa cells. These cells provide the oocyte essential nutrients for later stages: maturation, fertilization, and embryo development [10]. CCs primary function is to regulate the mechanism of oocyte meiotic arrest and resumption [11]. Maintaining the viability of the CCs is critical for oocyte maturation after vitrification. It has been reported that CCs viability is affected after vitrification [3], reducing oocyte maturation. Different types of cell communication have been described, such as tight junctions, desmosomes, cell adhesion molecules, and gap junction intercellular communication (GJIC) [12]. The latter establishes communication between CCs-oocyte and consists of arrays of intercellular channels that allow the exchange by passive diffusion of compounds < 1 KDa as metabolites, ions, sugars, second messengers, and water [13]. Gap junctions are composed of membrane hemichannels called connexons, each of them composed of six transmembrane protein subunits called connexins. GJIC is directly involved in oocyte meiotic arrest and resumption providing a direct communication by which cGMP enters the oocyte, inhibiting phosphodiesterase A, maintaining high levels of cAMP, and the meiotic arrest [14]. For resumption, LH receptor activation in the CCs reduces the influx of cGMP by causing the GJIC closure and meiotic resumption in mouse [15] and pigs [14]. Previous studies indicate that the integrity of the GJIC between CCs-oocyte during the first 4 h of maturation is crucial to resume meiosis properly [14, 16–18]. Also, GJIC is important for the regulation of chromatin remodeling and transcription during the first hours of bovine oocyte maturation [19]. The knowledge of the possible mechanisms involved in reducing GV oocyte maturation after vitrification is important for the development of more efficient vitrification protocols. Therefore, the aim of this study was to evaluate the GJIC integrity after the vitrification of immature porcine cumulus-oocyte complexes (COCs).

## Experimental design

Fresh COCs were randomly distributed in three groups: untreated (control), toxicity (cryoprotectants exposure), and vitrification, then subjected to in vitro maturation (IVM). Before and after IVM, at 0 h and 44 h, oocyte viability was measured in all groups. For viability, at least three replicates were performed with *n* = 162 control, *n* = 107 toxicity, and *n* = 128 vitrification, evaluated oocytes. For maturation, seven experiments were performed with *n* = 250 control, *n* = 175 toxicity, and *n* = 175 vitrification, evaluated oocytes. GJIC integrity was evaluated at 4, 8, 22, and 44 h of culture. Experiments were carried out in triplicate with *n* = 80 control, *n* = 80 carbenoxolone (positive control), *n* = 60 toxicity, and *n* = 80 vitrification, evaluated oocytes.

## Methods

Unless otherwise mentioned, all chemicals were acquired from Sigma Chemical Co. (St. Louis, MO, USA). For oocyte collection and IVM, ovaries were obtained from pre-pubertal Landrace gilts at a local slaughterhouse ¨Los Arcos¨, located in Texcoco, State of Mexico (animal health federal law authorization 6265375) and transported to the laboratory in a 0.9% NaCl solution at 25 °C within 2 h after collection. Ovarian follicles between 3 and 6 mm in diameter were punctured to obtain COCs. For COCs collection and washing, Tyrode modified medium supplemented with 10 mM sodium lactate, 10 mM HEPES and 1 mg/mL polyvinyl alcohol (PVA) (TL-HEPES-PVA) was used. Only oocytes with uniform cytoplasm surrounded by a two-four-layer compact mass of CCs were selected. COCs were washed three times in 500 μL drops of maturation medium composed of TCM-199 with Earle’s salts and 26.2 mM sodium bicarbonate (In Vitro, Mexico City) and supplemented with 0.1% PVA, 3.05 mM D-glucose, 0.91 mM sodium pyruvate, 0.57 mM cysteine and 10 ng/mL EGF. For IVM, groups from 30 to 50 COCs were placed in each well of a four-well dish (Thermo-Scientific Nunc, Rochester NY) containing 500 μL of maturation medium supplemented with 0.5 μg/mL luteinizing hormone (LH) and 0.5 μg/mL follicle stimulating hormone (FSH) for 44 h [5]. Oocyte culture was performed under mineral oil and incubated at 38.5 °C in an atmosphere of 5% CO_2_ in air and humidity at saturation.

For COCs exposure to cryoprotectants before IVM, groups of eight to ten COCs were exposed to the highest CPAs concentration solution containing TCM-199, 16% dimethylsulphoxide (Me_2_SO), 16% ethylene glycol (EG) and 0.4 M sucrose at 38.5 °C for 1 min (toxicity group). Immediately, COCs were recovered and washed three times in TL-HEPES-PVA medium and transferred to TCM-199 medium for IVM. Exposure time and temperature were selected to make them comparable to values commonly used for oocyte vitrification protocols [5].

For COCs vitrification and warming, the medium used was TCM-199-HEPES supplemented with 0.5 mM L-glutamine and 0.1% PVA (VW medium). For vitrification, COCs were washed twice in VW medium and sequentially equilibrated in the first vitrification solution containing 7.5% Me_2_SO + 7.5% EG for 3 min, and 1 min in a second vitrification solution containing 16% Me_2_SO, 16% EG and 0.4 M sucrose. Then, groups of seven COCs were placed in a 2 μL drop of the second vitrification solution and loaded into the Cryolock device (Importadora Mexicana de Materiales para Reproduccion Asistida S. A. de C.V., Mexico) in less than 1 min. Then, the Cryolock device was immediately plunged horizontally into liquid nitrogen and stored during 30 min [5]. Warming was performed by the one-step method [20]. For COCs recovery, the Cryolock device was submerged vertically in a four-well dish containing 800 μL of VW medium supplemented with 0.13 M sucrose. Finally, COCs were incubated in VW medium for 5 min, then recovered and transferred to maturation medium.

Oocyte viability was measured at T 0 h = immediately after COCs collection, and at T 44 h = after IVM in all groups. Oocytes were stained with 100 μL of 0.5 mg/mL Thiazolyl blue (MTT) diluted in PBS. The MTT stain evaluates the metabolic activity of cells. NADPH-dependent cellular oxidoreductase enzymes are capable of reducing the tetrazolium dye MTT to formazan (reflecting purple coloration) as an indicator of viable cells. After 1 h, oocytes were analyzed under a light microscope (Zeiss Axiostar) and classified as viable (purple-stained) and non-viable (colorless). To evaluate IVM, oocytes were stained with 10 μg/mL bisbenzimide (Hoechst 33342) diluted in PBS for 45 min and evaluated using an epifluorescence microscope (Zeiss Axiostar) at 40 X magnification. Oocytes showing a GV, or in the metaphase I (MI) were considered immature, and those in the metaphase II (MII) with the first polar body as matured. After COCs cryoprotectants exposure and vitrification, GJIC transfer between CCs-oocyte was evaluated using the acetoxymethyl (AM) ester derivative of the fluorescent indicator calcein (calcein-AM; Molecular Probes, Eugene, OR) method [14]. After 4, 8, 22 and 44 h of culture, COCs from all groups were exposed to calcein-AM for 15 min, then were transferred to calcein-AM free media and cultured for 25 min, allowing the dye exchange between CCs-oocyte. In the oocyte cytoplasm, nonspecific endogenous esterases hydrolyze the acetoxy-methyl groups to produce calcein (MW 622.54), a negatively charged molecule that is unable to permeate through the plasma membrane and can only pass from the CCs to the oocyte cytoplasm through GJIC, producing a fluorescent green coloration. Then, COCs were washed once in 0.01% (w/v) PVA and PBS and were analyzed under a confocal epifluorescence microscope (Zeiss, LSM T-PMT) with an argon laser (488 nm) at 60 X magnification to obtain pictures. Fluorescence intensity (FI) profiles of calcein in CCs-oocytes were obtained using the Zen blue lite 2.3 software. Lines were drawn through the COCs optical sections generated by the confocal microscope. Carbenoxolone, a gap junction blocker, was used at 100 μM as a positive control [21].

## Statistical analysis

Statistical analyses were carried out using GraphPad Prism 8.2.1 (GraphPad Software Inc.). The percentage of oocyte viability, maturation, and FI in control, toxicity, and vitrification groups were analyzed by a one-way ANOVA followed by Bonferroni multiple comparison test with a confidence level of *P* < 0.05. Data are presented as mean ± SD.

## Results

**Fig. 1:**
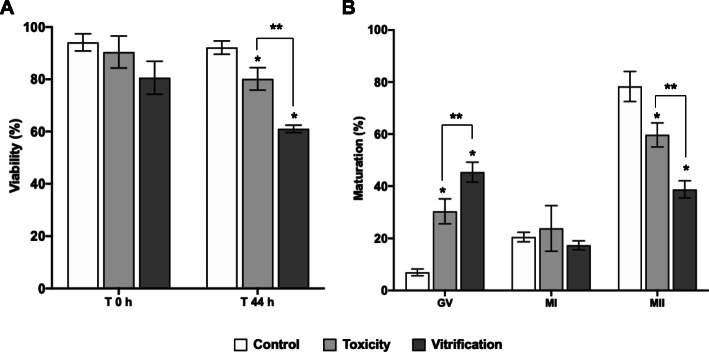
Effect of vitrification on oocyte viability and in vitro maturation. **a** Percentage of oocyte viability at T 0 h (immediately after vitrification) and after 44 h of culture. Oocyte viability decreased after 44 h of culture in the toxicity and vitrification groups. **b** Percentage of oocytes in different developmental stages after 44 h of culture. Oocyte maturation (MII stage) decreased in toxicity and vitrification groups. GV = germinal vesicle; MI = metaphase I; MII = metaphase II. Bars show the mean ± SD. * Significant differences vs. control. ** Significant differences toxicity vs. vitrification

**Fig. 2:**
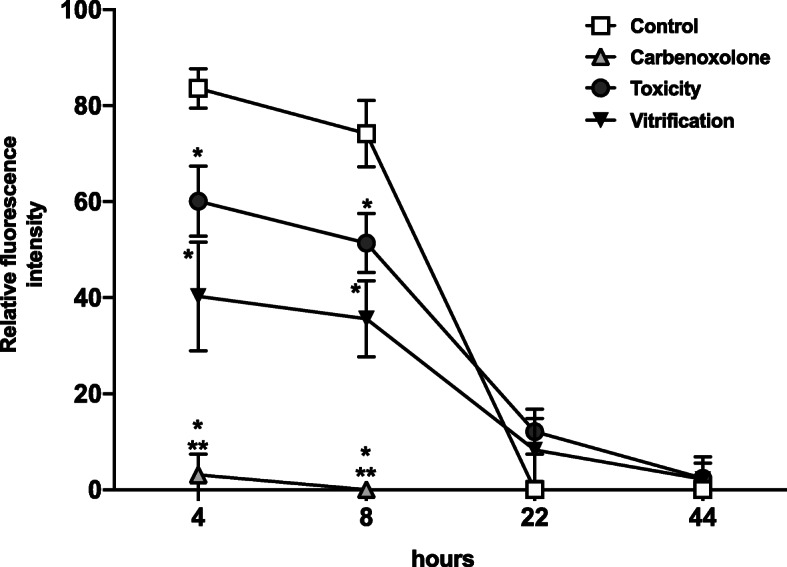
Effect of vitrification on GJIC in COCs during in vitro maturation. GJIC was measured in COCs after 4, 8, 22, and 44 h of culture by quantitative RFI. Lines were drawn through the COCs optical sections generated by the confocal microscope. Carbenoxolone was used as a positive control of GJIC blockage. The RFI values were converted to a scale of 1–100 for graphic representation. After cryoprotectants exposure (toxicity) and vitrification, GJIC decreased at 4 and 8 h of culture. GJIC = gap junction intercellular communication; COCs = cumulus-oocyte complexes; RFI = relative fluorescence intensity. Bars show the mean ± SD. * Significant differences vs. control. ** Significant differences vitrification vs. carbenoxolone

**Fig. 3:**
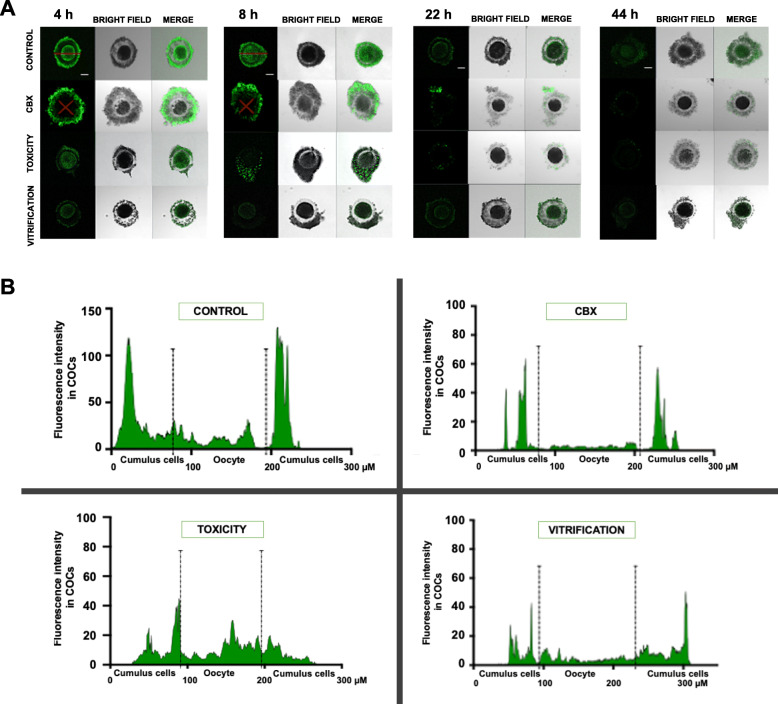
Representative images of GJIC evaluation after COCs vitrification. **a** Cumulus cell-oocyte GJIC was measured by the quantitative FI profiles of calcein in COCs. Lines were drawn through the COCs optical sections generated by the confocal microscope (red line). In the control group, high FI was observed in the CCs and oocyte at 4 and 8 h (red lines). Reduced FI was observed in carbenoxolone, toxicity, and vitrification groups after 4 and 8 h. Carbenoxolone was used as a positive control of GJIC blockage (red crossed lines). At 22 and 44 h of culture, reduced FI was observed in all groups. **b** Representative FI profiles in COCs from all groups. Graphs show the FI profiles of calcein (marked in green) transferred from the CCs to the oocyte. GJIC = gap junction intercellular communication; FI = fluorescence intensity. Scale bar = 30 μM

Oocyte viability at T 0 h was not statistically different from control (*P* > 0.05). However, cryoprotectants exposure and vitrification significantly decreased oocyte viability after 44 h of culture compared to control (*P* < 0.05), as shown in Fig. 1a. After vitrification, the MI stage was not affected compared to control. However, a significantly high percentage of GV oocytes was observed after cryoprotectants exposure and vitrification compared to control. Consequently, the percentage of MII oocytes significantly decreased after cryoprotectants exposure and vitrification compared to control. Also, the percentage of oocyte maturation was significantly lower in the vitrification group compared to the toxicity group (Fig. 1b). The evaluation of GJIC was performed after 4, 8, 22, and 44 h of culture, and it was expressed as relative fluorescence intensity (RFI), as shown in Fig. 2. In control, significantly high RFI was observed at 4 and 8 h, which was decreased at 22 and 44 h. After cryoprotectants exposure and vitrification, significantly reduced RFI was observed in COCs at 4 and 8 h compared to control. At 22 and 44 h, reduced or absent RFI was observed in all groups. Also significantly reduced RFI was observed in the vitrification group compared to the toxicity group (Fig. 2). Representative images of GJIC evaluation after vitrification are shown in Fig. 3. CCs-oocyte GJIC was measured by the quantitative FI profiles of calcein in COCs (Fig. 3a). In control, results showed that high FI is observed in the CCs-oocyte at 4 and 8 h (red lines). In the carbenoxolone group (a gap junction blocker), high FI was observed in the CCs but not in the oocyte at 4 and 8 h (red crossed lines). In the toxicity and vitrification groups, reduced FI was observed in the CCs-oocyte at 4 and 8 h. Also, in control and vitrification groups, reduced FI was observed in CCs-oocyte at 22 and 44 h (Fig. 3a). For all groups, FI profiles were measured in COCs using the confocal microscope software, as shown in Fig. 3b.

## Discussion and conclusion

The present study demonstrates that porcine immature oocyte viability decreased after 44 h of culture. These results confirm those previously reported [22], where the viability of pig oocytes decreases after IVM. This could be explained by the fact that during in vitro culture, the levels of O_2_ [23] and reactive oxygen species increase [24], affecting cell viability. Likewise, in the GV stage, oocytes are highly cryo-sensitive during the vitrification process compared to other developmental stages. Porcine oocytes vitrified in the GV stage, present a high intracellular lipid content, which prevents the permeability and protection capacity of intracellular cryoprotectants. Therefore, a high amount of intracellular lipids affects glutathione levels in vitrified oocytes, which increases the production of H_2_O_2_ and the generation of oxidative stress [25]. Also, GV oocyte meiotic spindle organization, chromosome and actin filaments distribution has been reported to be extremely sensitive to low temperatures, compared with MII oocytes [26]. Our results demonstrate that oocyte maturation was significantly affected after vitrification. However, the percentage of MI oocytes was not different. This may be due to the fact that some oocytes were able to break the GV, but remained arrested in MI without progressing to MII, where a significant decrease in oocyte maturation was observed.

GJIC is directly involved in oocyte meiotic arrest and resumption. In the present study, we evaluated the integrity of GJIC after vitrification using the calcein-AM transfer assay. As far as we know, information about the integrity of GJIC after the vitrification of porcine GV oocytes is limited. In porcine, using transmission electron microscopy, a study reported that gap junctions and microvilli were ruptured after GV oocyte vitrification [27]. Another study evaluated the transzonal projections (TZPs), by marking F-actin, as an indicator of gap junctions integrity. They observed the formation of large clusters of intracellular F-actin in vitrified oocytes [2]. Also, in feline GV oocytes, GJIC was evaluated by the lucifer yellow dye microinjection. They reported that vitrification by slow freezing or vitrification impairs intercellular junctions, which resulted in low oocyte maturation (32.5 and 14.1%, respectively) [28]. It has been reported that during the first hours of maturation (4–8 h) the functionality of the GJIC between the CCs-oocyte is crucial for the mechanism of meiotic resumption [14]. Domínguez et al. (2019) [18] reported that porcine oocyte exposure to perfluorodecanoic acid (PFDA) affected GJIC at 4.5 h of IVM suggesting that the inhibition of oocyte maturation by PFDA might be explained by the disruption of GJIC during the first 18 h of IVM. In the present study, during the first hours of culture, vitrified oocytes displayed lower FI, affecting GJIC. It was also observed that the functionality of the GJIC decreased from 22 to 44 h of IVM in the control. Appeltant et al. (2017) [2] reported that at 0 h the TZPs are present but at 20 h they are no longer observed. Some studies point out the possible relationship between the disruption of the GJIC during the first hours of culture with a decrease in IVM rates [17, 18]. It has been reported that through gap junctions, metabolites and signaling molecules are exchanged between the cumulus cells and the oocyte during the first 18 h, and subsequently decrease producing the rupture of the GV [14]. It has also been reported that cell communication between these cells during the first hours of IVM in bovine oocytes plays an important role in the regulation of chromatin remodeling and transcription [19].

Our results showed that vitrification impairs GJIC between the CCs-oocyte, which could explain the low rates of IVM obtained. GJIC functionality was observed in control oocytes at 4 and 8 h, in order to promote meiotic resumption. It was reported that GJIC functionality is critical during the first 4.5 h of IVM for meiotic resumption [16–18]. However, GJIC was significantly compromised after vitrification compared to control. Therefore, this study demonstrates that porcine oocyte viability and maturation after 44 h of culture decreased after vitrification. GJIC was also affected during the first hours of culture after the vitrification of immature oocytes, being one of the possible mechanisms by which oocyte maturation decreased.

## Data Availability

Not applicable.
